# Correction of ankle valgus by hemiepiphysiodesis using the tension band principle in patients with multiple hereditary exostosis

**DOI:** 10.1007/s11832-016-0742-8

**Published:** 2016-05-27

**Authors:** M. van Oosterbos, A. L. van der Zwan, H. J. van der Woude, S. J. Ham

**Affiliations:** Department of Orthopaedic Surgery, Onze Lieve Vrouwe Gasthuis, P.O. Box 95500, 1090 HM Amsterdam, The Netherlands; Department of Radiology, Onze Lieve Vrouwe Gasthuis, Amsterdam, The Netherlands

**Keywords:** Pediatric patient, Ankle vargus, Multiple hereditary exostoses, Eight-plate system, Temporary hemiepiphyseodesis

## Abstract

**Background:**

Ankle valgus is a common deformity in patients with multiple hereditary exostoses (MHE) and a potential risk factor for early degenerative arthritis. In children, medial hemiepiphysiodesis of the distal tibia is a relatively simple surgical technique used to correct this deformity. We present here the first results of applying this procedure using the eight-Plate guided growth system (eight-Plate) for growth guidance.

**Methods:**

Between 2006 and 2011 we performed hemiepiphysiodesis of the distal medial tibia in 30 ankles of 18 children with MHE using the eight-Plate. Weight-bearing total leg radiographs were obtained preoperatively, during follow-up and at the time of implant removal or when the distal tibial physis had closed. The lateral distal tibia angle (LDTA) was measured and fibular shortening assessed using the Malhotra classification. To evaluate the effect of hemiepiphysiodesis, we correlated the LDTA with age.

**Results:**

The mean age at time of surgery was 12.6 (range 9.5–15.0) years, and the mean preoperative LDTA was 76.9° (range 68.5°–83.5°). During follow-up, the implant was removed in 12 extremities and the physis had closed in 18 extremities. The mean LDTA at the time of implant removal or at closure of the physis was 83.6° (range 76.5°–90.0°). Mean correction of LDTA was 6.9° after a mean follow-up period of 22 (range 3–43) months. During follow-up, no changes in the Malhotra classification were found in any of the patients. Correction of the valgus deformity of the ankle was significantly correlated (*r* = −0.506) (*p* = 0.004) with age in all patients.

**Conclusion:**

Temporary medial hemiepiphyseodesis of the distal tibia seems to be an effective strategy for correcting ankle valgus in children with MHE. Timing of the intervention is, however, of importance. Hemiepiphyseodesis alone has no effect on the Malhotra classification.

**Level of evidence:**

IV, retrospective review.

## Introduction

Osteochondromas are the most common bone tumors in children and adolescents. These are defined as cartilage-capped bony outgrowths that are broad-based or stemmed and made up of cortex and a marrow cavity, both of which are continuous with the host bone. Osteochondromas arise from the metaphyseal ends of rapidly growing long bones, as well as from the axial skeleton [1, 2], and they account for approximately 40 % of all benign bone tumors [3]. The actual prevalence of these tumors in the general population is unknown since many patients remain asymptomatic and are never seen by a physician. On the basis of the number of patients who do seek treatment, 90 % of these tumors occur as a solitary osteochondroma, whereas the remaining 10 % are multiple osteochondromas. The latter, also known as multiple hereditary exostoses (MHE), is an autosomal dominant disorder that is frequently associated with characteristic skeletal deformities, including valgus deformity of knees and ankles [4].

Valgus deformity of the ankle is common in MHE patients, being present in approximately 50 % of patients with MHE [5–7]. Valgus deformity results from underdevelopment of the lateral tibial physis, leading to relative overgrowth at the medial side, thus forcing the ankle into a valgus configuration [5]. In general, milder cases of MHE show little or no associated osteochondromatous changes at the ankle region. The more pronounced cases are usually, but not always, associated with multiple osteochondromas located around the malleoli and especially at the lower tibiofibular junction. These valgus deformities may result in activity-related pain, limited ankle function and early degenerative arthritis [8].

Surgical techniques currently used to manage this deformity include temporary hemiepiphyseodesis of the distal tibia with the use of a screw or staple in growing children and possible supramalleolar correction–osteotomy of the tibia, with or without fibular lengthening, in the adolescent or adult patients [2, 9, 10]. The technique of guiding the growth by hemiepiphyseodesis with a plate fixed with two screws serving as a tension band was introduced in 2004, with good clinical results in the proximal and distal tibia and distal femur [11–13]. This system of fixation does not compress the physis like a staple, and it has a better bony fixation [11, 12].

Since August 2006, we have used the eight-Plate guided growth system (eight-plate) in medial hemiepiphyseodesis of the distal tibia for valgus deformity in patients with MHE. The first results of this procedure are reported here.

## Materials and methods

### Patients

The medical records of all patients with MHE who had been treated in our center, a tertiary referral center for MHE in the Netherlands, were reviewed. Between August 2006 and August 2011, hemiepiphysiodesis using the eight-Plate (Orthofix Srl, Verona, Italy) was performed in 28 consecutive pediatric patients (18 boys, 10 girls) with valgus deformity of the ankle. The surgical procedure was performed bilaterally in 17 patients and unilaterally in 11 patients. The total number of ankles treated was 45. The indication for surgery had been correction and/or prevention of excessive valgus deformity of the ankle in children in order to prevent the development of early degenerative arthritis [8]. At the time of surgery, all children included in the study had open distal growth plates of both the tibia and fibula.

The final patient population for analysis consisted of 18 patients with 30 involved ankles who were considered to have completed treatment because either the implant had been removed (12 ankles) during follow-up or the distal tibial physis had closed as a result of skeletal maturity during follow-up (18 ankles). Treatment was not complete in the remaining nine patients, and these patients were therefore excluded from this analysis.

All patients were treated by a single surgeon employing a standardized surgical technique as reported by Stevens [12]. Through an approximately 3-cm incision, the surgeon placed a single plate on the medial distal tibia, taking care to preserve the saphenous vein and the periosteum. The smallest, green colored 12-mm plate of the eight-Plate system was used in all cases, as were the smallest epiphyseal and metaphyseal purple-colored 4.5-mm-diameter and 16-mm-long screws. In ten cases, additional surgical procedures were performed in the same extremity together with the hemiepiphysiodesis of the distal medial tibia, including excision of nine osteochondromas located on proximal tibia or distal femur, as well as hemiepiphyseodesis of the proximal tibia in eight extremities and of the distal femur in two extremities. Fluoroscopy was used to verify adequate placement of the hardware in both the anteroposterior and lateral plane in all cases. Following the surgery, full weight bearing and return to normal activities was tolerated and encouraged as soon as possible. Most patients used crutches for only the first postoperative days, and a restriction on sports activities was advised for the first 4 weeks following surgery. All patients were seen at the outpatient clinic on a regular basis, and anterior–posterior weight-bearing total leg radiographs were performed every 4–6 months until the implant was removed or skeletal maturity had been reached. Patient data were included for this analysis when the implant had been removed or the distal tibial physis had closed.

### Radiographic analysis

Ankle valgus was measured on anterior–posterior weight-bearing total leg radiographs. All radiographs were obtained digitally using a picture achieving communication system (PACS). All measurements were made by the first author and the senior author, with agreement on the measured angles and values.

The lateral distal tibia angle (LDTA) was measured between the mid-diaphyseal line of the tibia and the line parallel to the distal tibial plafond [14] (Fig. 1). The published range for the LDTA is 86°–92°. Fibular shortening was classified using the Malhotra classification [15] (Fig. 2). In the Malhotra classification, grade 0 indicates that the distal fibular physis is at the level of the tibia plafond; grade 1, that the fibular physis is at the same level as the tibial epiphysis; grade 2, that both the fibular and tibial physis are at the same level; grade 3, that the fibular physis is proximal to the tibial physis and triangular tibial physis with delayed ossification of the lateral portion. Figure 3 shows a radiograph obtained immediately following surgery and one obtained during follow-up. Prior to this study the intraobserver reliability of LDTA was determined by calculating the standard error of the mean after measuring the LDTA of 35 ankles twice with an interval of 1 month (SEM 1.3).

**Fig. 1:**
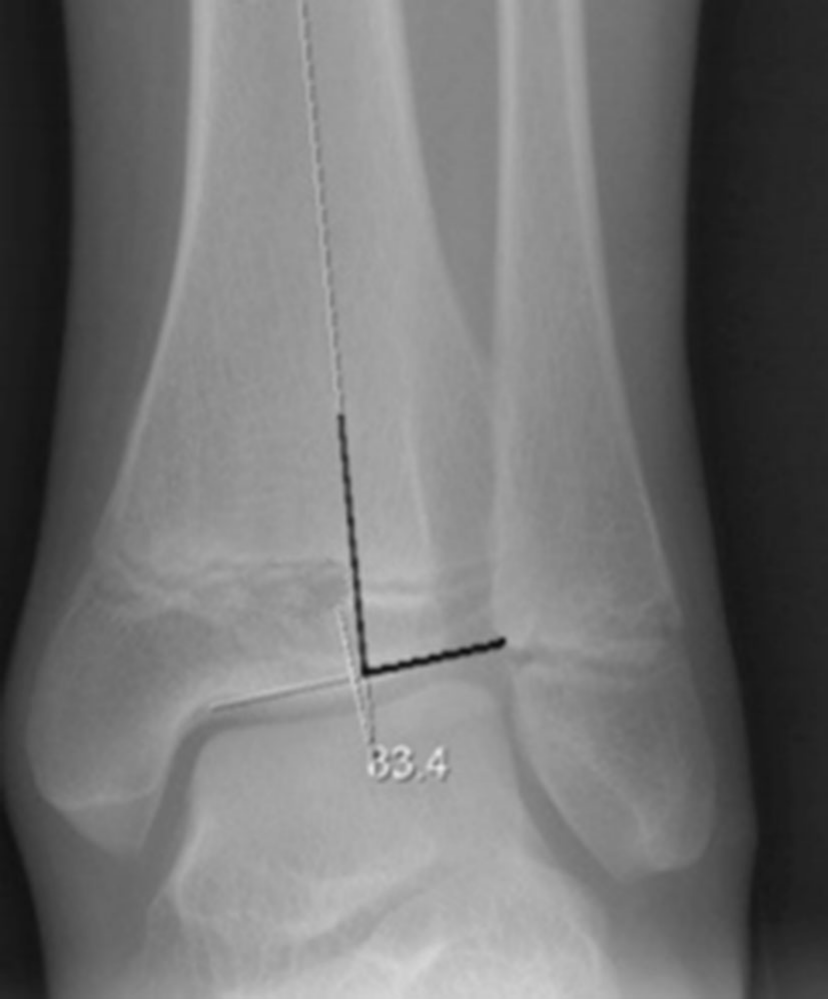
Lateral distal tibia angle (LDTA) is measured between the mid-diaphyseal line of the tibia and the line parallel to the distal tibial plafond

**Fig. 2:**
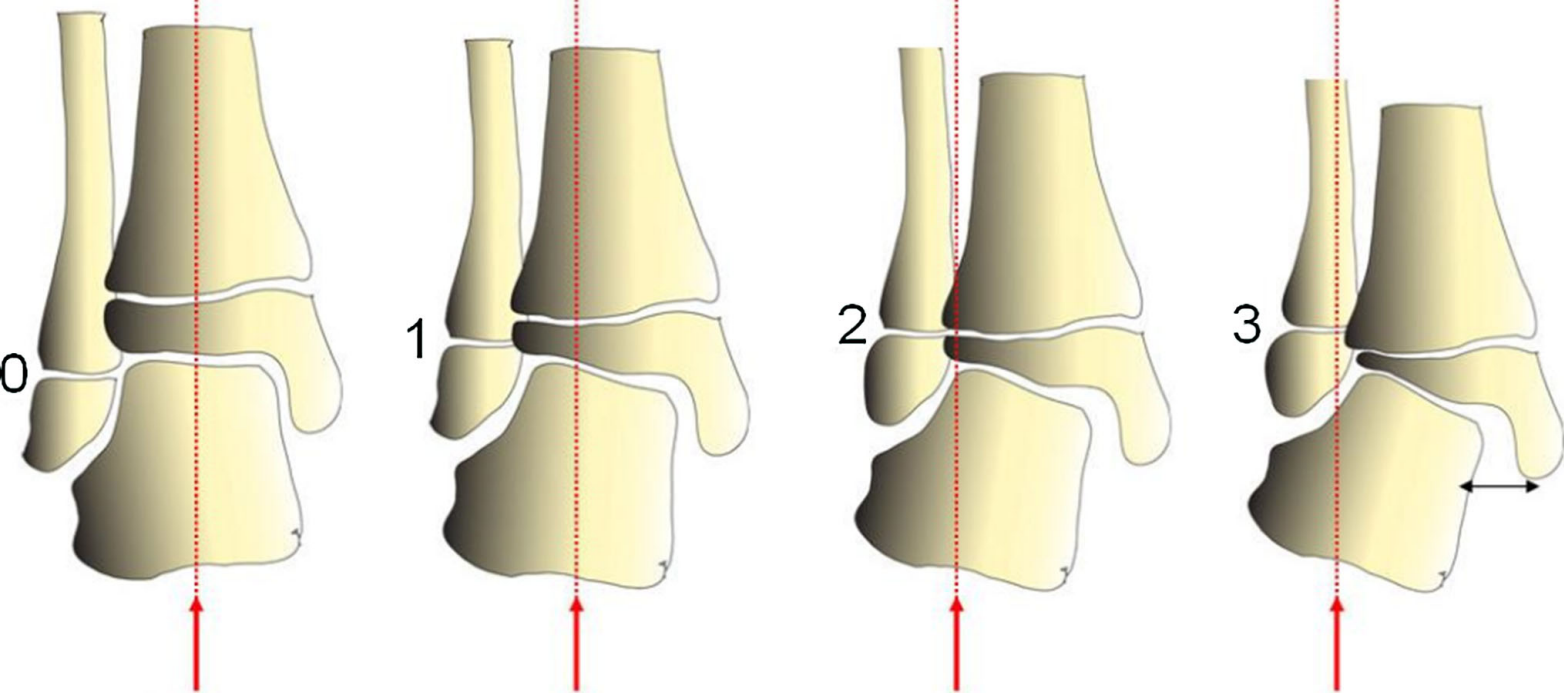
Malhotra classification of ankle valgus. *0*, normal state with the fibular physis at the level of the plafond, *1*–*3* states of progressive valgus, as indicated by the proximal position of the fibular physis (*red dotted lines* and* arrows*). Image reprinted with permission from *Medscape* *Drugs & Diseases*

**Fig. 3:**
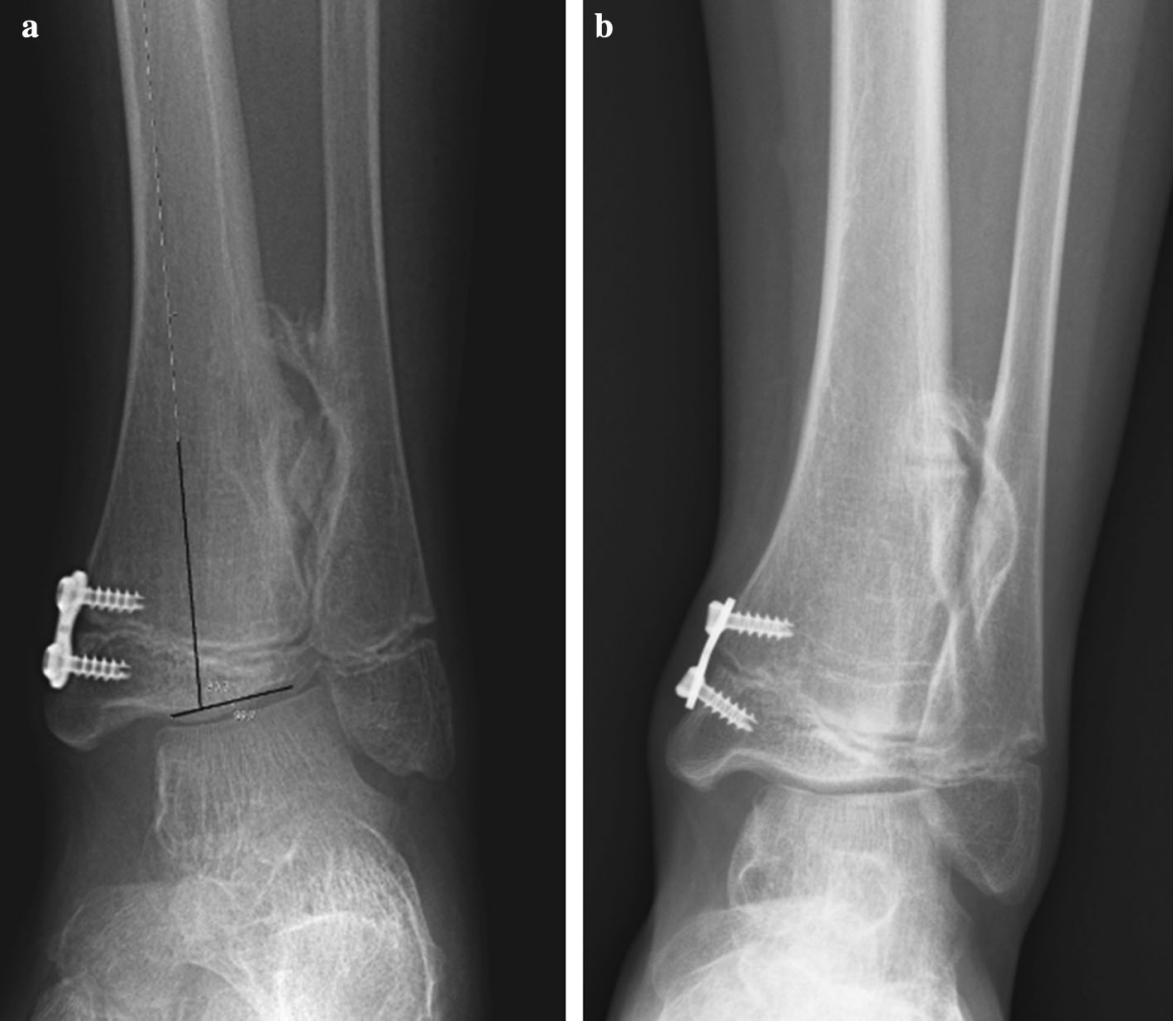
Radiograph obtained immediately following surgery (**a**) and later during follow-up (**b**)

## Statistics

Values are expressed as the mean and range (minimum–maximum). Data were analyzed using a statistical software program (SPSS ver. 19; IBM Corp., Armonk, NY). Pearson’s correlation coefficient was obtained to determine whether correction of the valgus deformity was significantly correlated with age. A value of *p* < 0.05 was used as the threshold for statistical significance.

## Results

### Patients

The study population consisted of 18 children (10 boys, 8 girls) with an overall mean age at the time of the initial procedure of 12.6 (range 9.5–15) years. The mean age of the boys and girls at the time of the initial procedure was 13.1 (range 11.9–15.0) and 11.8 (range 9.5–13.3) years, respectively. The mean overall follow-up period until removal of the implant or total physeal closure, as evidence on radiographs, was 22 (range 3–40) months. The mean follow-up for boys and girls was 27 (range 11–45) and 22 (range 10–40) months, respectively. No intraoperative or postoperative complications occurred in this series of patients.

### Radiographic evaluation

The mean preoperative LDTA of the 30 ankles analyzed was 76.9° (range 68.5°–83.5°). At the time of implant removal or physeal closure, the mean LDTA was 83.6° (range 76.5°–90°). The mean LDTA correction, therefore, was 6.9° (range 1°–16.5°). Full correction, considered to be an LDTA of 86°–90°, was reached in eight extremities. The correction of the LDTA was nearly equal in boys and girls, being 7.1° after a mean follow-up of 22 (range 3–42) months and 6.5° after a mean follow-up of 21 (range 15–40) months, respectively. None of the patient’s ankles overcorrected in a varus deformity. No tibiofibular synostosis was seen either during the preoperative period or during follow-up examinations.

Correction of the valgus deformity was significantly correlated with age at the time of hemiepiphysiodesis, with younger patients having the greatest correction (*r* = −0.506; *p* = 0.004) (Table 1).

**Table 1:** Patient data

Patient number	Sex	Age at surgery (years)	Side	Closed physis at SR	Length of FU after SR (months)	Presurgery LDTA (°)	LDTA after FU (°)	Correction (°)	Correction/month (°)
1	Female	13.3	R	Yes	18	79	88	9	0.50
		13.3	L	Yes	18	81.5	90	8.5	0.47
2	Male	12.0	R	No	11	76	83	7	0.64
		11.3	L	No	14	78	89	11	0.79
3	Male	13.3	R	Yes	19	81	85	4	0.21
		13.3	L	Yes	19	78	83.5	5.5	0.29
4	Male	14.7	L	No	3	78.5	81	2.5	0.83
5	Female	11.6	L	Yes	37	80	85	5	0.14
6	Female	10.9	R	No	17	80	87.5	7.5	0.44
7	Female	11.2	R	No	16	78	85	7	0.44
		11.2	L	Yes	40	72	82	10	0.25
8	Female	12.8	R	Yes	11	78.5	81.5	3	0.27
		12.8	L	Yes	11	79	82.5	3.5	0.32
9	Male	12.0	R	No	32	72	82.5	10.5	0.33
		12.0	L	No	32	74.5	85	10.5	0.33
10	Male	13.8	R	Yes	28	70	77.5	7.5	0.27
		13.8	L	Yes	28	74	81	7	0.25
11	Male	15.0	R	Yes	10	74	78	4	0.40
		15.0	L	Yes	10	75.5	76.5	1	0.10
12	Male	12.6	R	Yes	33	68.5	79.5	11	0.33
		12.6	L	Yes	33	78.5	87.5	9	0.27
13	Male	12.1	R	No	35	72	81	9	0.26
		12.1	L	No	42	68.5	85	16.5	0.39
14	Male	14.7	R	Yes	14	80	83.5	3.5	0.25
		14.7	L	Yes	14	80.5	81	0.5	0.04
15	Male	12.0	R	No	17	75	85	10	0.59
		12.0	L	No	17	81	87	6	0.35
16	Female	11.5	L	Yes	32	83.5	89	6.5	0.20
17	Female	12.3	L	Yes	18	80.5	85	4.5	0.25
18	Female	9.5	L	No	15	80	87.5	7.5	0.50

*R* Right,* L* left, *SR* screw removal, *FU* follow-up,* LDTA* lateral distal tibia angle

Figure 4 shows the average LDTA correction (in degrees) in the boys of our patient population according to age at the time of surgery. A similar graph could not be constructed for girls due to the limited number of extremities.

**Fig. 4:**
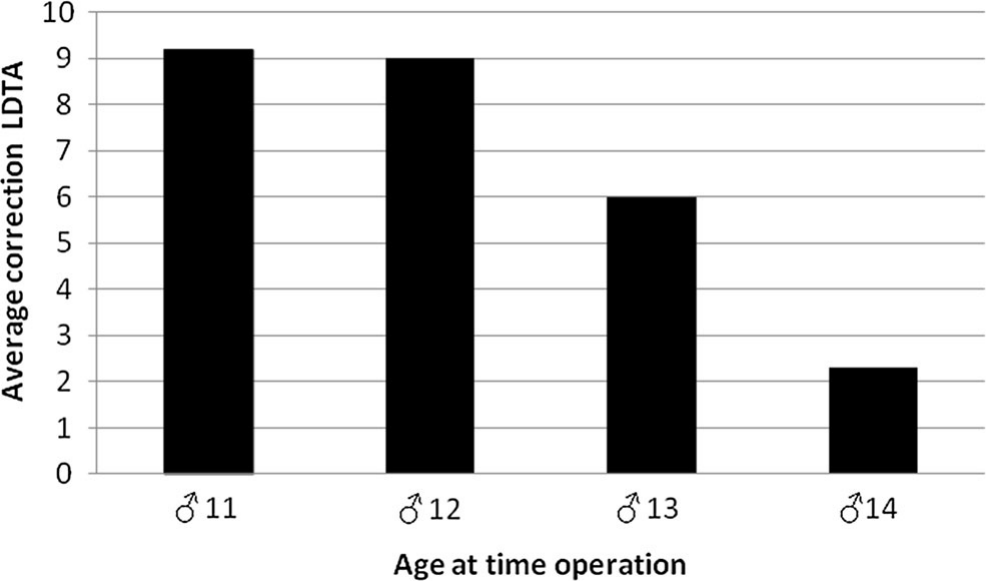
Mean LDTA correction (in degrees) in the boys of this patient series according to age (in years) at the time of surgery

The preoperative Malhotra classification was: Malhotra grade 0 for five ankles, Malhotra grade 1 for 16 ankles and Malhotra grade 2 for nine ankles. Comparison of the first radiographs with measurements with the radiographs made at the time of plate removal or closure of the distal tibial physis revealed that no significant change in Malhotra classification had occurred.

## Discussion

In MHE, valgus deformity of the ankle is seen in approximately 50 % of affected persons [5–7, 16]. Osteochondromas of the tibia, fibula, or both are often present in the deformed ankles, as is, occasionally, tibiofibular synostosis, most notable after cessation of growth. The etiology of ankle valgus in children with MHE is poorly understood. Growth abnormalities may result in relative shortening of the fibula, fibular bowing, tapering of the lateral aspect of the distal tibial epiphysis, and valgus tilt of the ankle [5, 7, 10, 17].

Indications for surgery in children with valgus deformity of the ankle include pain due to local pressure and irritation, abnormal mechanical alignment, decreased ankle range of motion (ROM), cosmetic factors, and (possible) prevention of early degenerative osteoarthritis [8].

The prevention of early degenerative osteoarthritis is an important primary goal of surgery in children with ankle valgus. Noonan et al. [8] found signs of early osteoarthritis in 19 % of the evaluated ankle joints of formerly untreated adult MHE patients who were evaluated in a natural history study. When present, signs of arthritic changes, based on radiographic evaluation, ranged from mild joint space narrowing to severe degenerative osteoarthritis. The measured values of ankle valgus (in degrees) were significantly greater in patients with radiographic evidence of degenerative arthritis, and patients with osteoarthritic changes also had significantly decreased ROM of the ankle joint. In this patient series [8], approximately 50 % of subjects reported some form of ankle discomfort when walking distances of ≥1 mile. Given the fact that the average age of the subjects in this study was only 42 years, higher rates of degenerative arthritis might possibly occur with longer periods of follow-up. Noonan et al. [8] concluded that prophylactic surgical procedures in children that were designed to improve alignment in the ankle joint might be justified due to the high rate of early osteoarthritis in adults with MHE.

Ozaki et al. [16] studied 21 patients with MHE and found a valgus deformity of the ankle in 15 (71 %) of these, with 29 of the 42 ankles (69 %) affected [16]. In one-half of the patients, the valgus deformity progressed during growth. These authors reported that isolated excision of distal osteochondromas had no effect on valgus deformity.

In growing children, surgical treatment options for correction of (excessive) ankle valgus include medial hemiepiphyseodesis with a staple or transepiphyseal screw. The aim of the hemiepiphysiodesis procedure should be prevention of early osteoarthritis and reduction of the need for more extensive and invasive surgery at a later age. In full-grown children and adults, fibular lengthening and supramalleolar osteotomy with the use of plate fixation or a ring fixator are surgical options when the correction is considered to be mandatory [2, 10]. To our knowledge, our study is the first to report the treatment of valgus deformities of the ankle joint in growing children with MHE using the eight-Plate for hemiepiphysiodesis. We started to use this device in the present series of patients with ankle vargus following earlier application of the eight-Plate in angular deformities of the knee, with the results leading to the conclusion that the device was as effective as staples, correction was predictable, and the correction rate was related to the age of the patient. Previous studies comparing the use of an eight-Plate with staples reported that the surgical time was significantly shorter for procedures with the eight-Plate and the complication rate was similar in both techniques, with no relevant difference in the number and severity of complications [18, 19].

For ankle valgus, Rupprecht et al. [9] reviewed nine children with MHE (15 ankles; 13 boys and 2 girls) who had been treated with temporary screw epiphyseodesis of the distal tibia to correct ankle valgus. Mean age at the time of surgery was 11.8 (range 9.6–14.7) years, and the mean preoperative tibiotalar tilt was 14.3° (comparable LDTA 75.1°). The average correction achieved was 13.9° after a mean follow-up of 36 months. In our series, the mean correction achieved was 6.9° after a mean follow-up of 25 months. The patients in our series were on average 8 months older than those in the series of Rupprecht et al. [9], and the treatment time in our study was 14 months shorter.

Stevens et al. [13] reviewed 33 children (57 ankles) who had been treated with temporary eight-Plate epiphyseodesis of the distal tibia to correct valgus deformities. In their patient series, valgus deformities were caused by a variety of underlying etiologies, with only one patient with MHE included. Mean age at surgery was 10.4 years, and the mean LDTA improved from 78.7° prior to surgery to 90° at implant removal. Mean correction at implant removal was 11.3° after a mean follow-up of 27 months. Stevens et al. [13] also observed that younger patients tended to have greater improvement of correction. This tendency is in agreement with our observations on our patient series as we also found that correction of valgus deformity was significantly correlated with the age of the patients. The mean age of the patients in our series was 2.2 years older than that of the patients in the series of Stevens et al. [13].

Our results show that, in general, hemiepiphysiodesis of the distal medial tibia should be performed at a younger age than that of the patients in the present series in order to achieve full correction of the LDTA. However, hemiepiphysiodesis performed in patients with MHE at too early age could result in recurrence of ankle valgus following hardware removal, as has been reported by Dricroll et al. [20]. This was not a problem in our series as most of the distal tibial growth plates (18 ankles) had already closed during follow-up. Furthermore, as the valgus deformity in patients with MHE could be the result of asymmetrical growth disturbance of the epiphyseal plate, which is a characteristic of MHE, optimal timing of the surgical intervention in such patients could differ from that in patients with ankle valgus attributable to idiopathic causes [21]. The development of an algorithm for the optimal timing of surgery in both boys and girls with ankle valgus due to MHE will require data on a substantial higher number of patients than included in the present patient series, but it is the goal of future research by our group. It is believed that screws in the distal physis of the tibia can influence the joint line symmetry, which in turn might induce a deformity; it has also been stated that longer screws might prevent this deformity. Both of these factors associated with the use of screws will also be investigated by our group in future studies. Consistent with previous investigators, we found that the Malhotra classification did not significantly change after treatment [15, 22–24]. In our opinion, this finding supports the theory that eight-Plate hemiepiphyseodesis achieves its effect by tethering the medial aspect of the distal tibial physis and that neither the fibular station nor the epiphyseal wedging is altered by this technique [25] The major limitations of this study are its retrospective design, the relatively small series, and the absence of a comparison group.

In conclusion, temporary medial hemiepiphyseodesis of the distal tibia seems to be an effective method to correct ankle valgus in children with MHE. The treatment procedure is technically simple with low morbidity. Hemiepiphyseodesis alone has no effect on the Malhotra classification. Timing of the intervention is of utmost importance. Future research will be focused on larger series of patients, the introduction of an algorithm for the optimal timing of hemiepiphysiodesis in this location, and the influence of other variables, such as presence and size of adjacent osteochondromas, on hemiepiphysiodesis.
